# Undernutrition Among Pregnant Women in an Urban Municipality in Ghana: A Cross-Sectional Study

**DOI:** 10.1155/jnme/4420685

**Published:** 2025-02-07

**Authors:** Agartha Afful Boateng, Dorothy Serwaa Boakye, Charles Owusu-Aduomi Botchwey, Richard Boateng, Emmanuel Kumah

**Affiliations:** Department of Health Administration and Education, University of Education, Winneba, Central Region, Ghana

**Keywords:** cross-sectional study, dietary diversity, pregnant women, prevalence, undernutrition

## Abstract

**Introduction:** Despite efforts to address malnutrition, it remains prevalent in Ghana and other developing nations. High rates of malnutrition hinder the achievement of the health-related sustainable development goals. Understanding the factors contributing to undernutrition among pregnant women (PW) is essential for developing targeted interventions. This study, therefore, aims to assess the prevalence of undernutrition, dietary diversity (DD), and predictors of undernutrition among PW in Effutu Municipality in the Central Region of Ghana.

**Methods:** Two hospitals within the Effutu Municipality were recruited for the study. The study was conducted among 301 PW (15–49 years) recruited through simple random sampling from September 20, 2022, to October 24, 2022. The mid-upper arm circumference and minimum DD score for women were used as a proxy for undernutrition and DD, respectively. SPSS version 26 was used for data analysis. Descriptive statistics, bivariate analysis, and binary regression analysis were conducted to describe variables and identify the association between dependent and independent variables.

**Results:** The majority of the respondents (40.5%) were in the age range of 26–30 years and were single (53.2%). The prevalence of undernutrition was 9.3%, while 7.6% of the respondents had poor DD scores. Factors identified to be associated with undernutrition were DD score (OR = 15.244, 95% CI: 5.399–43.040), employment status (OR = 3.311, 95% CI: 1.075–10.195), and parity (OR = 2.903, 95% CI: 1.125–7.492).

**Conclusion:** Effutu Municipality in Ghana faces a moderate prevalence of undernutrition among PW, despite generally adequate DD. Targeted interventions focusing on improving DD, particularly among unemployed and multiparous women, are essential for addressing undernutrition and improving maternal and fetal health outcomes in the study area. These findings underscore the importance of context-specific strategies to combat malnutrition among PW.

## 1. Introduction

The global burden of malnutrition, encompassing both underweight and overweight or obesity, affects an estimated 462 million individuals and 1.9 million adults, respectively, with females, infants, children, and adolescents being particularly vulnerable [1]. Notably, undernutrition prevails more than twice as much as overnutrition globally [1]. During pregnancy, undernutrition not only jeopardizes the health of the mother but also adversely impacts the fetus [2]. Maternal undernutrition can lead to complications such as iron-deficiency anemia, hemorrhage, pre-eclampsia, and maternal mortality [2, 3], while fetal health risks include iodine deficiency leading to cretinism, folate deficiency causing neural tube defects, and vitamin D deficiency predisposing the baby to rickets, low birth weight, and stillbirth [2]. These noncommunicable diseases strain resource-limited healthcare systems in developing nations, hindering the achievement of sustainable development goals (SDGs) 5 and 2, aimed at reducing global maternal mortality rates to 70 per 100,000 live births and addressing all forms of malnutrition by 2030 [2]. Undernutrition contributes significantly to maternal mortality [4]. Promoting dietary diversity (DD) among pregnant women (PW) and continuously assessing undernutrition among PW are crucial interventions to improve pregnancy outcomes [4].

Common proxies for evaluating DD and undernutrition among women of reproductive age include the minimum DD score for women (MDD-W) and mid-upper arm circumference (MUAC), respectively [5–7]. The MUAC serves as an inexpensive and noninvasive method to assess undernutrition among PW, particularly in resource-limited settings, with studies indicating its association with DD [7, 8]. Consequently, women with adequate DD scores are likely to exhibit acceptable MUAC values, positively impacting pregnancy outcomes. Therefore, numerous studies advocate for PW to enhance their DD to prevent undernutrition and improve pregnancy outcomes [8, 9].

In a cross-sectional quantitative study conducted in the Ashanti Region of Ghana utilizing the MUAC to assess malnutrition, the authors found that 10.6% of respondents suffered from undernutrition [10]. Although factors such as marital status, maternal age, and iron intake were significantly associated with maternal malnutrition using a chi-square test, these factors did not predict the MUAC during binary logistic regression analysis. However, the study did not explore the association between maternal undernutrition and DD. Similarly, a study among pregnant adolescents in the Ashanti Region using MDD-W to assess DD found that 56% of the respondents had inadequate DD scores [11]. Nonetheless, the researchers did not report any association between undernutrition and DD scores, and the study exclusively focused on pregnant adolescents. Another study among PW in the Northern Region of Ghana reported a 26% prevalence of undernutrition among respondents, with factors such as household food security, maternal age, and level of education predicting undernutrition but not DD [12]. These studies suggest inter-regional variation in undernutrition prevalence and associated factors among PW in Ghana.

The Effutu Municipality, situated in the Central Region of Ghana, faces a stunting prevalence of 35.5% among children under five [13, 14], exceeding the regional rate of 34%. Child nutritional status correlates with maternal status during and after pregnancy [15, 16], with children born to undernourished mothers exhibiting a 13% higher prevalence of undernutrition [15]. Given the high prevalence of stunting among under-fives in Effutu Municipality, it is reasonable to infer the existence of undernutrition among PW in the area. However, no study has specifically investigated undernutrition and DD among PW in this locality. Moreover, maternal socioeconomic status, early pregnancy, maternal age, level of education, multiple pregnancies, and DD are associated with maternal nutritional status [6, 8, 12], with variations observed across communities in Ghana, potentially explaining differences in undernutrition prevalence and associated factors nationwide. Therefore, empirical data are warranted to inform context-specific interventions aimed at addressing undernutrition among PW in communities and achieving SDG goal 2. Hence, this study aims to assess the prevalence of undernutrition, DD, and predictors of undernutrition among PW in Effutu Municipality, Central Region, Ghana.

## 2. Materials and Methods

### 2.1. Study Design and Setting

This quantitative cross-sectional survey was conducted between September 20, 2022, and October 24, 2022, in Effutu Municipality, located in the Central Region of Ghana. The study focused on two hospitals: Winneba Municipal Hospital, a publicly funded institution, and Otoo Memorial Hospital, a privately owned primary healthcare facility. Winneba Municipal Hospital's antenatal clinic typically sees an average of 150 PW per month, although this number fluctuates. Meanwhile, Otoo Memorial Hospital's antenatal clinic serves approximately 100 PW monthly. These facilities were purposefully chosen due to their status as primary healthcare providers situated in urban areas, offering antenatal services to a diverse range of women from various socioeconomic backgrounds within the municipality. This selection ensured a suitable pool of respondents for the study. The recruitment of participants occurred at the antenatal clinics of both hospitals before they received healthcare services. However, data collection took place after they had undergone their healthcare appointments.

### 2.2. Study Population

The study population consisted of PW aged 15–49 years who had resided in the Effutu Municipality for at least six months prior to data collection, attended antenatal care at selected healthcare facilities, were pregnant with a single baby, and expressed willingness to participate. Exclusions from the study encompassed women with multiple pregnancies, those experiencing illness or hospitalization, those who declined participation, and women with severe hyperemesis gravidarum who were admitted to the hospital and unable to fill out the questionnaire due to their ill health were excluded from the study. This decision was based on the consideration that their condition could interfere with their ability to participate fully in the data collection process. Women with mild to moderate hyperemesis gravidarum were included in the study, as their participation was without significant limitations.

### 2.3. Sample Size Determination and Sampling Procedure

The sample size for this study was determined using the Cochran formula [17], with parameters set at a 95% confidence level, a 5% margin of error, and a 26% prevalence of malnutrition among PW, as reported by Saaka et al. [12]. Factoring in a 10% nonresponse rate, the final sample size was calculated to be 326.

Quota sampling was employed, considering that the government hospital typically sees around 150 antenatal care visits per month, while the private hospital attends to approximately 100 such visits monthly. Consequently, 196 respondents were selected from the government facility and 130 from the private-owned facility.

The following outlines the calculation of the quota sample of the two facilities:

(1) $$\begin{array}{c} \text{GPH}=\frac{n}{N}\times e, \end{array}$$

where

• GPH is the quota per hospital,
• *n* represents the number of antenatal care services provided per month by each facility,
• *N* is the total antenatal care provided per month across both facilities, and
• *e* is the desired sample size for the study.

For the government hospital,

• *n* = 150,
• *N* = 250 (which is 100 from the private hospital + 150 from the government hospital),
• *e* = 326.

Thus, the GPH for the government hospital is

(2) $$\begin{array}{c} \text{GPH}=\frac{150}{250}\times 326=196. \end{array}$$

For the private hospital,

• *n* = 100,
• *N* = 250,
• *e* = 326.

The GPH for the private hospital is

(3) $$\begin{array}{c} \text{GPH}=\frac{100}{250}\times 326=130. \end{array}$$

At the selected facilities, simple random sampling was conducted. A lottery system was utilized: pieces of paper marked with “Yes” or “No” (326 of each) were mixed in a container, from which the PW were asked to draw with replacement. Those who drew “Yes” were recruited for the study, and in cases where a respondent selected “Yes” but declined participation, the “Yes” was replaced, continuing until the desired sample size was attained. Of the 326 respondents who selected “Yes,” 301 agreed to participate, resulting in a response rate of 92.3%. Only 25 respondents, 16 from the government hospital and 9 from the private facility, declined to participate.

### 2.4. Study Variables and Measures

#### 2.4.1. Prevalence of Undernutrition

The prevalence of undernutrition was determined utilizing the MUAC, chosen for its simplicity and cost-effectiveness in assessing nutritional status [18]. Additionally, MUAC measurement is noninvasive, increasing respondent acceptance. Using a standard nonelastic tape, the MUAC was measured at the midpoint between the shoulder tip (olecranon process) and elbow tip (acromion process) of the left arm, rounded to the nearest 0.1 cm with the arm unclothed. The MUAC was categorized dichotomously, with measurements below 23 cm indicating undernutrition and 23 cm or above indicating normal nutritional status [8, 19, 20].

#### 2.4.2. DD

DD was evaluated through a 24-h dietary recall, employing the MDD-W. This entailed respondents recalling all foods consumed from midnight to midnight the previous day. Food items were tailored to the local context by incorporating regional substitutes. The questionnaire comprised 10 food groups: starchy staples (grains, roots, tubers, and plantains), pulses, nuts and seeds, dairy, meat, poultry, fish, eggs, dark leafy greens and vegetables, other vitamin A–rich fruits and vegetables, other vegetables, and other fruits. Respondents reporting consumption from a group (*Yes*) received a score of 1, while those not consumed (*No*) scored 0. Scores were summed, ranging from 0 to 10 [11]. Women consuming at least 5 out of the 10 food groups within the 24-h period were categorized as having good DD (scores 5–10), whereas those consuming fewer than five groups were deemed to have poor DD (scores 0–4) [5].

#### 2.4.3. Sociodemographic Characteristics

Data on sociodemographic characteristics were collected using a pretested, structured questionnaire. Variables included age, marital status, education level, family size, employment status, parity, and gestational age.

### 2.5. Data Collection and Analysis

The study data were gathered through a designed questionnaire, informed by the existing literature [5, 18, 19]. It comprised three sections: (A) evaluating DD, (B) assessing the prevalence of malnutrition, and (C) gathering sociodemographic data. Originally in English, the questionnaire was translated into the local languages of “Twi” or “Fante” for respondents unable to read English. The process involved professional translators fluent in both languages, followed by a meticulous back-translation step to verify conceptual equivalence. Additionally, experts in the field reviewed the translated questionnaire to confirm content validity, ensuring that the questions accurately captured the intended constructs across languages.

Two trained research assistants conducted the data collection. They underwent a comprehensive training session on the instrument and participated in its pretesting. Throughout the fieldwork, the first author along with two other authors reviewed the completed questionnaires for accuracy. Additionally, the research assistants conducted onsite checks for completeness, making necessary corrections as needed.

Data collection took place in a consulting room at the antenatal clinic postreceiving care, with each respondent's session lasting approximately 15–20 min. No financial incentives were provided to respondents.

Data analysis employed SPSS version 26. Chi-square tests were utilized to explore associations between undernutrition (the outcome variable), DD, and sociodemographic characteristics of the respondents. Significant variables (*p* value < 0.05) from the chi-square analysis were subjected to binary regression analysis to identify the predictors of undernutrition among PW. The results were reported using odds ratios (ORs) with 95% confidence intervals (CIs). A significance level of *p* < 0.05 and a CI of 95% were set for the study. Descriptive statistics, such as frequencies and percentages, were also utilized in presenting the study's findings.

### 2.6. Reliability Test

Internal consistency using the Cronbach alpha (*α*) coefficient was used to assess the reliability of subscales in this study. The Cronbach alpha (*α*) coefficient for DD with 10 items was 0.788, indicating that this subscale was reliable [21]. Since the MUAC consisted of only one item, reliability was not tested for this variable.

### 2.7. Ethical Consideration and Informed Consent

This study adhered to the principles outlined in the Declaration of Helsinki [22] and followed the guidelines for research set forth by the University of Education, Winneba. Ethical approval was obtained from the University of Education Winneba Ethics and Research Committee with Reference No. UEWC/28. Permission was sought from the selected facilities prior to data collection. Throughout the data collection process, every effort was made to ensure that respondents participated voluntarily and without coercion. All study participants provided their consent by either signing or thumb-printing a consent form. The anonymity and confidentiality of respondents were rigorously maintained throughout the study. Completed questionnaires were securely stored in lockable cardboard boxes in the first author's office. Electronic databases were password protected and accessible only to the research team.

## 3. Results

### 3.1. Sociodemographic Characteristics of the Respondents

Out of the 326 administered questionnaires, 301 were returned, resulting in a response rate of 92.3%. A significant proportion of the respondents (40.5%) fell within the 26–30 age bracket and were unmarried (53.2%). Moreover, a large majority had received a basic level of education (68.1%), was currently employed (89.7%), and had between 0 and 3 children (72.4%). Further elaboration on these results is provided in Table 1.

### 3.2. DD Score

The study findings revealed that, within the 24 h preceding the study, the primary food groups consumed by the respondents were predominantly starchy staples (92.0%), followed by other vitamin A–rich fruits (88.7%), flesh foods (86.7%), and other fruits (84.4%). Conversely, the least consumed foods during this period were beans and peas (26.2%), vitamin A–rich dark green leafy vegetables (24.1%), and nuts and seeds (20.3%). Notably, 92.4% of the respondents had consumed foods from at least five food groups, while 7.4% had not, as depicted in Table 2.

### 3.3. The Prevalence of Undernutrition

**Figure 1:**
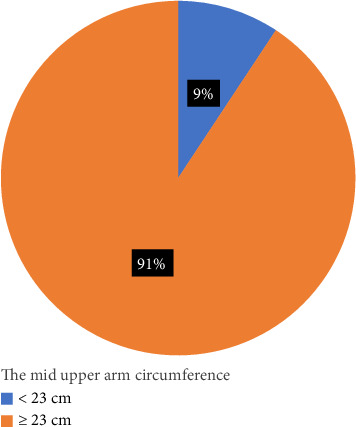
The prevalence of undernutrition.

Figure 1 shows the MUAC measurement used to determine the prevalence of undernutrition among the respondents. About 9% (9.3%) of the PW obtained a measurement below 23 cm indicating undernutrition and 90.7% obtained 23 cm and above indicating normal nutritional status. Thus, the prevalence of undernutrition among the PW was 9.3%.

### 3.4. Predictors of Undernutrition

The bivariate results of the study, using a chi-square test, revealed that there was a statistically significant association between the DD score and the prevalence of undernutrition (*p* ≤ 0.001). The sociodemographic characteristics associated with the prevalence of undernutrition were employment status (*p* ≤ 0.001) and parity (*p* ≤ 0.001). The rest of the sociodemographic characteristics did not show any significant statistical association with undernutrition among PW (*p* >  0.05).  Table 3 provides the details of the findings.

The results of the binary regression analysis showed that the independent predictors of undernutrition among PW were DD score (*p* ≤ 0.001), employment status (*p* ≤ 0.037), and parity (*p* ≤ 0.028). Undernutrition was more than 15 times (OR = 15.244, 95% CI: 5.399–43.040) and 3 times (OR = 3.311, 95% CI: 1.075–10.195) more likely to be prevalent among PW with poor DD and the unemployed than those with good DD, and the employed, respectively. Undernutrition was also almost three times (OR = 2.903, 95% CI: 1.125–7.492) more likely to be prevalent among PW with more than 3 parity than those with less than or equal to 3 parity. Details of these findings are presented in Table 4

(4) $$\begin{array}{c} {\text{Nagelkerke }R}^{2}=30.4\%. \end{array}$$

## 4. Discussion

This study aimed at assessing the prevalence of undernutrition, predictors, and DD among PW in Effutu Municipality in the Central Region of Ghana.

Our findings indicate that among the study respondents, 9.3% were undernourished, as defined by a MUAC measurement of < 23 cm. This prevalence, although higher than the national prevalence of 3.6%, is lower compared to the 10.6% found by Ayensu et al. [10] using a MUAC cutoff of < 25 cm in both rural and urban Ghanaian communities. Similarly, a study conducted in Dessie town, northeastern Ethiopia, reported a prevalence of undernutrition of 19.5% (MUAC < 23 cm), more than twice the prevalence observed in our study [8]. Likewise, in Khartoum, Sudan, a study [19] setting MUAC at < 23 cm reported a prevalence of undernutrition of 12.5%, higher than our finding. In southwest Ethiopia, Nigatu and Gemeda [7] identified a prevalence of undernutrition of 28.6% (MUAC < 21 cm), indicating significant regional variations.

These differences in prevalence may be attributed to geographical and socioeconomic disparities across study settings. In Ghana, regions such as Greater Accra, housing the capital city with predominantly urban populations, tend to exhibit lower prevalence rates of undernutrition compared to northern regions [2]. Effutu Municipality, with approximately 90% of its population residing in urban areas, likely experiences lower rates of undernutrition, possibly due to better access to nutritious foods [11]. Urban residents, particularly females, often have access to diverse and readily available food options, contributing to improve DD compared to rural counterparts.

Moreover, discrepancies in prevalence findings may stem from variations in MUAC cutoff points used across studies, highlighting the need for standardized criteria for assessing the MUAC among PW. The adoption of internationally accepted MUAC cutoff points is crucial for enhancing the accuracy and comparability of malnutrition assessments among PW. Standardized measurement criteria would allow for more consistent cross-study comparisons and better global monitoring of maternal malnutrition. Intersectorial collaborations are essential to establish such standards and facilitate research consistency within this special population.

Regarding DD, the majority (92.4%) of our respondents met the minimum DD requirements for women, surpassing the rates reported in other studies conducted in different settings [10–12, 23]. This high DD may be attributed to factors such as educational attainment and access to nutritional education, particularly through antenatal care services. Nutritional education integrated into antenatal care programs in Ghana likely contributes to improved dietary practices among PW.

Our study also identified predictors for undernutrition among PW, including DD, employment status, and parity. Poor DD was associated with a higher prevalence of undernutrition, consistent with findings from previous research [2, 8]. Employment status emerged as a significant factor, with employed women exhibiting better DD, likely due to increased purchasing power for varied foods. Additionally, women with higher parity showed a higher prevalence of undernutrition, highlighting the importance of nutritional support for women with multiple pregnancies to meet their increased nutrient requirements.

It is important to acknowledge the notably high OR for DD (OR = 15.244, CI: 5.399–43.040). While we adjusted for key variables such as age, education, income level, and gestational age, the possibility of unmeasured confounding variables cannot be entirely ruled out. Factors such as physical activity levels, prepregnancy BMI, or specific health conditions were not included in our model and may have influenced the results. These unaccounted-for confounders could partially explain the magnitude of the observed association between DD and the outcomes measured in this study. Future research should consider a more comprehensive set of variables to ensure a more robust understanding of the relationships identified.

Addressing undernutrition among PW requires a multifaceted approach, including promoting girl child education, equitable access to antenatal care services, and empowering women economically. Continuous nutritional education and the provision of free nutritional supplements are essential to meet the unique nutritional needs of PW, particularly those with multiple pregnancies.

## 5. Strength and Limitation

The findings from our study are particularly relevant to the local context, as they reflect the unique nutritional and healthcare challenges faced by PW in this region. The study provides valuable insights that can inform targeted interventions aimed at improving maternal nutrition within similar communities. However, the study has some inherent limitations. We recognize that the study's generalizability may be limited due to the focus on a specific geographic area and population. As such, the results may not fully apply to other regions with different socioeconomic, cultural, and healthcare contexts. There may be some response bias and/or recall bias as DD was self-reported by the respondents based on the previous day's 24-h recall. The study also recruited only PW between the ages of 15–49* *years in the Effutu Municipality of the Central Region of Ghana. A key limitation of this study is the potential for sampling bias due to the use of quota sampling from only two hospitals (Winneba Municipal and Otoo Memorial) based on antenatal clinic attendance. This approach may exclude PW who seek care at other facilities or do not attend these hospitals, leading to a sample that may not fully represent the wider population. As a result, the findings may have limited external validity and may not be generalizable to all PW in the municipality. Another limitation of this study is the potential for residual confounding. While adjustments were made for several important variables, other potential confounders, such as household income, physical activity, prepregnancy BMI, and health conditions, were not included in the model. As a result, the high OR observed for DD may be influenced by these unmeasured factors. Future studies should incorporate a broader range of variables to better control for potential confounding and provide a clearer understanding of the relationship between DD and health outcomes.

## 6. Conclusion

Undernutrition prevails among PW in the study area, a concern demanding immediate public health action due to its subtle yet profound impact on both maternal and fetal health. The prevalence found is more than twice the national prevalence (3.6%) of undernutrition among PW. Factors such as the diversity of a PW's diet, employment status, and parity significantly influence the occurrence of malnutrition. Thus, continuous nutritional education is essential to foster dietary variety and promote the socioeconomic empowerment of women through gainful employment. Future studies should more explicitly assess the relationship between gestational age and nutritional status to provide a more comprehensive understanding of how these needs evolve during pregnancy.

## Figures and Tables

**Table 1:** Sociodemographic characteristics of studied respondents.

Variable	Categories	Frequency	Percentages (%)
Age in years	15–20	50	16.5
	21–25	82	27.1
	26–30	122	40.5
	31–35	37	12.2
	36+	10	3.7

Marital status			
Single	Never married	73	24.3
	Widow	11	2.0
	Divorced	6	3.7
	Cohabiting	70	23.3
Married	Married	141	46.8

Level of education	No formal educ.	21	7.0
	Basic education	205	68.1
	SHS	65	21.6
	Tertiary	10	3.3

Ethnicity			
Akans	Akans	151	50.2
Others	Others	150	49.8

Employment status			
Employed	Pro/tech/mana	72	23.9
	Sales and services	42	14.0
	Unskilled work	129	42.9
	Agriculture	27	9.0
Unemployed	Unemployed	31	10.3

Family size	1–5	134	44.5
	5+	167	55.5

Gestational age	1–3 months	81	26.9
	4–6 months	128	42.5
	7–9 months	92	30.6

Parity	0–3	218	72.4
	3+	83	27.6

*Note:* Pro/tech/mana represents the professionals/technicians/managers, which comprise nurses, teachers, hairdressers, seamstresses, nail technicians, shop owners, administrators, security personnel, and medical doctors. Basic education represents the primary and junior high school. SHS represents the senior high school. Others represent Ga-Adanbge, Ewe, Mole-Dagbani, Guan, Gurman, Grusi, Mande, and others.

**Table 2:** Dietary diversity among respondents.

Food category	Frequency	Percentage (%)
All starchy staple foods		
Yes	277	92.0
No	24	8.0
Beans and peas		
Yes	222	73.8
No	79	26.2
Nuts and seeds		
Yes	240	79.7
No	61	20.3
Diary and dairy products		
Yes	246	81.7
No	55	18.3
Flesh foods (meat and fish)		
Yes	261	86.7
No	40	13.3
Eggs		
Yes	254	84.4
No	47	15.6
Vitamin A–rich dark green leafy vegetables		
Yes	226	75.1
No	75	24.9
Other vitamin A–rich fruits		
Yes	267	88.7
No	34	11.3
Other vegetables		
Yes	252	83.7
No	49	16.3
Other fruits		
Yes	254	84.4
No	47	15.6
Consume at least 5 of the 10 food groups	278	92.4
Consume less than 5 of the 10 food groups	23	7.6

**Table 3:** Bivariate analysis of the relationship between undernutrition, and sociodemographic characteristics and dietary diversity.

Categories	Total	MUAC		Chi-square (*X*^2^)	*p* value
		< 23 cm (*n* = 28)	≥ 23 cm (*n* = 273)		
DD					
Poor	23	12 (52.2%)	11 (47.8%)	**54.25**	**0.001**
Good	273	16 (5.8%)	262 (94.2%)		
Age					
15–30	195	14 (7.2%)	181 (92.8%)	2.958	0.085
30+	105	14 (13.2%)	92 (86.8%)		
Marital status					
Married	141	11 (7.8%)	130 (92.2%)	0.708	0.400
Single	160	17 (10.6%)	143 (89.4%)		
Level of education					
No formal education	21	9 (42.9%)	12 (57.1%)	1.423	0.207
Basic education	205	10 (4.9%)	195 (95.1%)		
SHS/tertiary	75	9 (12.0%)	66 (88%)		
Employment status					
Unemployed	31	22 (71.0%)	9 (29.0%)	**15.945**	**0.001**
Employed	270	9 (7%)	251 (93%)		
Gestational age					
1st	81	9 (11.1%)	72 (88.9%)	1.364	0.505
2nd	128	9 (7.0%)	119 (93.0%)		
3rd	92	10 (10.9%)	82 (89.1%)		
Parity					
0–3	218	11 (5.0%)	207 (95.0%)	**16.977**	**0.00**
3+	83	66 (79.5%)	17 (20.5%)		
Ethnicity					
Akan	151	11 (7.3%)	140 (92.7%)	1.462	0.227
Others	150	17 (11.3%)	133 (88.7%)		

*Note:* The bold values indicate statistical significance of association.

**Table 4:** Binary regression results of independent predictors of undernutrition.

Model	Odd ratio	Sig	Confidence level	
			Upper bound	Lower bound
DDS				
Poor Good (ref)	15.244	**0.001**	5.399	43.040

Employment status				
Unemployment Employment (ref)	3.311	**0.037**	1.075	10.195

Parity				
3+ 0–3 (ref)	2.903	**0.028**	1.125	7.492

*Note:* The bold values indicate statistical significance of association.

Abbreviation: DDS = dietary diversity score.

## Data Availability

The data used in this analysis are available from the corresponding author on request.

## Nomenclature

PW: Pregnant women
DD: Dietary diversity
MUAC: Mid-upper arm circumference
